# Simultaneous molecular formula determinations of natural compounds in a plant extract using 15 T Fourier transform ion cyclotron resonance mass spectrometry

**DOI:** 10.1186/1746-4811-9-15

**Published:** 2013-05-30

**Authors:** Kyu Hwan Park, Min Sun Kim, Sun Jong Baek, Ik Hyun Bae, Sang-Wan Seo, Jongjin Kim, Yong Kook Shin, Yong-Moon Lee, Hyun Sik Kim

**Affiliations:** 1 Division of Mass Spectrometry Research, Korea Basic Science Institute, Ochang, Chungcheongbuk-Do 363-883, South Korea; 2 Bio Center, Chungbuk Techno Park, Ochang, Chungcheongbuk-Do 363-883, South Korea; 3 College of Pharmacy, Chungbuk National University, Cheongju 361-763, South Korea

**Keywords:** FT-ICR MS, Ultra-high resolution, Isotopic fine structure, Molecular formula determination, *Panax ginseng*, Ginsenosides

## Abstract

**Background:**

Plant extracts are a reservoir of pharmacologically active substances; however, conventional analytical methods can analyze only a small portion of an extract. Here, we report a high-throughput analytical method capable of determining most phytochemicals in a plant extract and of providing their molecular formulae from a single experiment using ultra-high-resolution electrospray ionization mass spectrometry (UHR ESI MS). UHR mass profiling was used to analyze natural compounds in a 70% ethanol ginseng extract, which was directly infused into a 15 T Fourier transform ion cyclotron resonance (FT-ICR) mass spectrometer for less than 10 min without a separation process.

**Results:**

The UHR FT-ICR MS yielded a mass accuracy of 0.5 ppm and a mass resolving power (m/Δm) of 1,000,000–270,000 for the range *m/z* 290–1,100. The mass resolution was sufficient to resolve the isotopic fine structure (IFS) of many compounds in the extract. After noise removal from 1,552 peaks, 405 compounds were detected. The molecular formulae of 123 compounds, including 33 ginsenosides, were determined using the observed IFS, exact monoisotopic mass, and exact mass difference. Liquid chromatography (LC)/FT-ICR MS of the extract was performed to compare the high-throughput performance of UHR ESI FT-ICR MS. The LC/FT-ICR MS detected only 129 compounds, including 19 ginsenosides. The result showed that UHR ESI FT-ICR MS identified three times more compounds than LC/FT-ICR MS and in a relatively shorter time. The molecular formula determination by UHR FT-ICR MS was validated by LC and tandem MS analyses of three known ginsenosides.

**Conclusions:**

UHR mass profiling of a plant extract by 15 T FT-ICR MS showed that multiple compounds were simultaneously detected and their molecular formulae were decisively determined by a single experiment with ultra-high mass resolution and mass accuracy. Simultaneous molecular determination of multiple natural products by UHR ESI FT-ICR MS would be a powerful method to profile a wide range of natural compounds.

## Background

Plant extracts contain a large number of components, including many pharmacologically active compounds. Numerous compounds in plant extracts can be beneficial in treating many diseases [1,2]; however, the complexity of the phytochemicals makes their analysis difficult and inhibits our understanding of the mechanisms that control their medicinal effects. There is no analytical method capable of evaluating all of the compounds present in a plant extract. Most analytical methods for plant extracts employ a combination of bioactivity assays and separation steps to isolate a few target compounds from a pool of numerous components. Although these traditional methods have been useful, there are disadvantages such as the high cost in time and labor, the blindness of molecular information, the possible loss of target compounds during the separation stage, and the disregarding of many active compounds not screened by the bio-assays used [3]. In general, separation methods use one or more molecular characteristics to discriminate compounds. Compounds that do not exhibit these characteristics are not separated properly or may even be lost during the separation process. For example, reversed-phase high-performance liquid chromatography (HPLC) employs hydrophobicity and seldom detects extremely hydrophilic or hydrophobic compounds such as petroleum and natural products, which can be analyzed by direct infusion into a mass spectrometer [4,5]. As separation-based methods can detect only some of the compounds extracted from a sample, there is a need for high-throughput (HT) analytical methods applicable for the rapid analysis of most compounds in a plant extract. Many studies to develop HT methods have focused on enhancing the peak capacity of HT screening to analyze a larger number of compounds. For example, multi-dimensional liquid chromatography [6,7] and high-resolution mass spectrometry (HR MS) [8,9] have been optimized in this fashion.

Fourier transform ion cyclotron resonance mass spectrometry (FT-ICR MS) is a common analytical method providing unparalleled resolution and sub-ppm accuracy in mass measurement [10-12]. Although FT-ICR MS in narrowband mode can achieve a mass resolving power at the level of several millions, narrowband mode is not suitable for investigating mixtures because of its narrow detection mass range in comparison with broadband mode. With the development of high-field FT-ICR MS, the resolution of FT-ICR MS in broadband mode has become high enough to resolve more than 5,000 species within a *m*/*z* range of 200–900 [13], and FT-ICR MS can simultaneously detect multiple ions to determine most compounds in a mixture without separation steps. These HT advantages of HR FT-ICR MS have been applied in studies of various mixtures such as metabolome [3,14,15], petroleome [13], lipidome [16,17], and herbalome [8,18] analyses. HR MS of a compound has been used to report probable molecular formula candidates. From previous studies, it is known that the molecular formula of a small organic molecule (less than 500 amu) can be determined, if the molecular mass is measured at 1 ppm accuracy together with its isotopic pattern [19]. In real sample analysis, the determination of a molecular formula by MS with a mass resolving power (m/Δm) of less than 100,000 is quite difficult due to isobaric compounds and adduct ions, which increase the number of candidate formulae within a mass window. With high field FT-ICR MS, the isotopic fine structure (IFS) has been revealed at ultra-high resolution (UHR) and used to decisively determine the molecular formula for small organic compounds [9,18,20].

The IFS of a single molecular ion produces a unique pattern of mass peaks owing to the different mass defects of isotopic contributions such as ^2^H, ^15^N, ^17^O, ^18^O, ^33^S, and ^34^S, as well as ^13^C, the main contributor to the isotopic pattern. Since the mass values and their intensities in the IFS of a molecule exactly reflect the atomic composition of the molecule, the IFS is a fingerprint of the molecular formula. Using IFS and high mass accuracy, fast and confirmative molecular formula determinations of multiple compounds in a mixture are possible in real sample analysis. Considering that the identification of compounds in HT analysis is generally achieved by matching with chemical databases, and only a small portion of the possible phytochemicals are registered in chemical databases, HT molecular formula determination by IFS would be very useful in plant extract studies.

**Figure 1:**
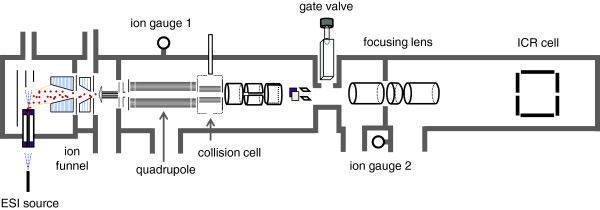
**Scheme of the 15 T FT-ICR mass spectrometer.** Ions introduced on the left side are focused and guided to the ICR detector positioned on the right side in the scheme. Several key compartments are indicated. The dimensions have been altered slightly for illustrative purposes.

In this study, an extract of ginseng was analyzed directly by UHR FT-ICR MS with a 15 T superconducting magnet to detect and determine the molecular formula of multiple compounds simultaneously. A scheme of the instrument is shown in Figure 1. Molecular formulae of more than 100 ginseng compounds were determined by their IFS. Liquid chromatography (LC)/FT-ICR MS of the ginseng extract was also performed, and the compounds detected by both MS approaches were compared to investigate the capability of UHR FT-ICR MS profiling in the study of phytochemicals.

## Results and discussion

### UHR ESI FT-ICR MS of ginseng extract

**Figure 2:**
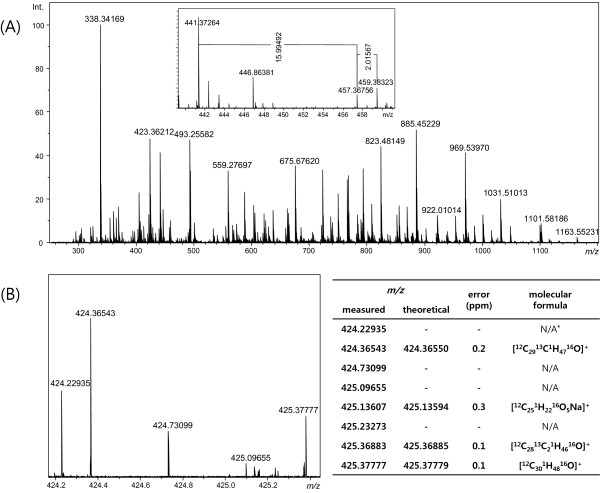
**UHR MS spectrum of ginseng extract.** (**A**) The UHR mass spectrum of ginseng extract obtained by a 15 T FT-ICR mass spectrometer. (**B**) The zoomed spectrum shows that the resolution was sufficient to resolve all peaks within a 1-amu span. The molecular formulae determined by IFS are listed in the side table.

The UHR mass profile of the compounds in a 70% ethanol ginseng extract was obtained using a 15 T FT-ICR mass spectrometer (Figure 2). The mass accuracy of the spectrum was 0.5 ppm after external calibration with 0.1 mg/mL arginine aqueous solution. The external calibration was performed on seven arginine cluster peaks in the mass range *m*/*z* 250–1500 with quadratic regression, and the maximum error was 0.47 ppm. The mass resolving power of the spectrum was 1,000,000–270,000 at the range of *m/z* 290–1,100. Within a single mass spectrum, 1552 peaks were detected with the peak-picking threshold at a signal to noise ratio (S/N) of 5. Because the detection of the M + 1 and M + 2 isotope peaks was difficult for low-abundance compounds, signals without corresponding M + 1 isotope peaks were regarded as noise. After removing the noise peaks, 405 compounds were detected in the extract by UHR electrospray ionization (ESI)/FT-ICR MS in positive ion mode. Although the mass resolution was not sufficient to clearly show the IFS of ions larger than *m/z* 850, the IFS of the chemical with *m*/*z* 985.5 was observed with a mass resolving power of 300,000 at *m*/*z* 1000, and the achieved mass resolution was sufficient to show the IFSs of molecules with *m*/*z* <850. The observed IFSs were used to determine the elemental compositions of corresponding chemicals with *m*/*z* <850. The zoomed spectrum in Figure 2B shows that the UHR of the spectrum resolves all of the observed peaks within 1 *m*/*z* unit. The assignment of a molecular formula to a peak was enabled by the high mass accuracy and the IFS revealed in the UHR mass spectrum as described later. The assigned molecular formulae determined by the experiment are listed in the side table (Figure 2B). The elemental compositions of several peaks in Figure 2B were not determined, because there was no candidate formula satisfying the 0.5 ppm mass tolerance for the limited elements. The unassigned peaks may contain elements such as F, S, P, halogens, or inorganic elements. Even though the peak at *m/z* 425.09655 was matched with [C_18_H_9_N_12_O_2_]^+^ (425.09659 amu) within a 0.1-ppm mass tolerance, the molecular formula was rejected, because the observed IFS of the peak was not compatible with the theoretical IFS of [C_18_H_9_N_12_O_2_]^+^. This result demonstrates that determining the IFS is crucial to avoid false positive assignment.

### Molecular formula determination

**Figure 3:**
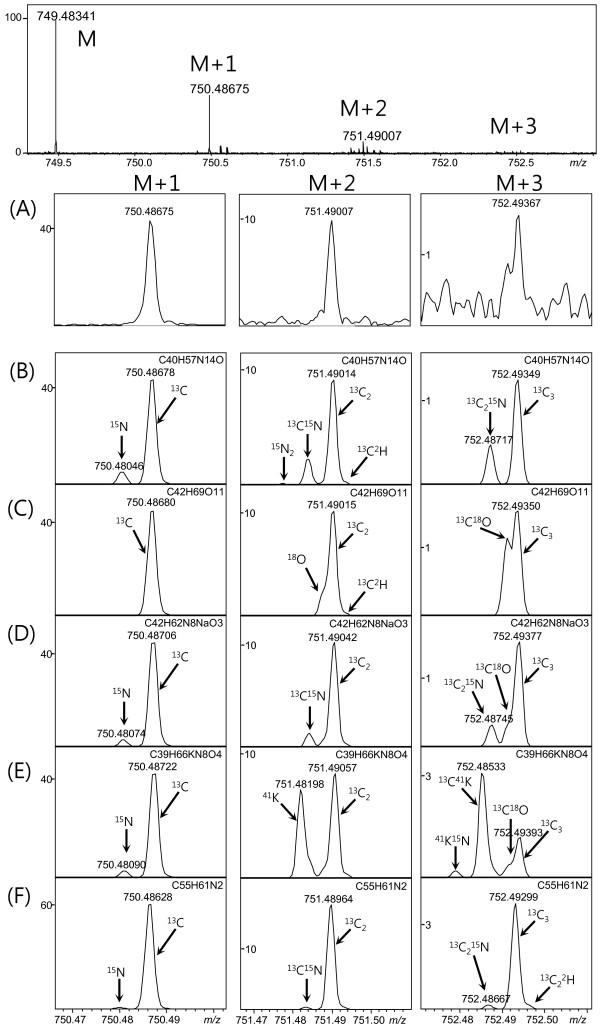
**Comparison of the experimental and the theoretical IFSs.** The experimental IFS of the ions with monoisotopic peaks at *m/z* 749.48341 (**A**) and five theoretical IFSs for [C_40_H_57_N_14_O]^+^ (**B**), [C_42_H_69_O_11_]^+^ (**C**), [C_42_H_62_N_8_O_3_Na]^+^ (**D**), [C_39_H_66_N_8_O_4_K]^+^ (**E**), and [C_55_H_61_N_2_]^+^ (**F**). From the comparison between the experimental IFS and the theoretical IFSs, the ionic formula of the compound at *m/z* 749.48341 was assigned to [C_42_H_69_O_11_]^+^, the molecular formula of (**C**). Calculated peak intensity of the each isotopic peak in the isotopic fine structures of candidate molecular ions is listed in Additional file 1.

The molecular formula of a natural compound was determined by comparing the isotope pattern observed experimentally with the theoretical IFS and the monoisotopic peak calculated by the Generate Molecular Formula (GMF) software tool. Since the maximum deviation of measured mass values in the spectrum (Figure 2A) was 0.5 ppm, the mass tolerance of GMF for candidate generation was also set to 0.5 ppm. GMF was applied to a monoisotopic peak to generate candidate formulae and their theoretical IFSs. The high mass accuracy of the spectrum considerably reduced the number of possible molecular formulae for each peak, especially for compounds whose molecular weights were typically less than 1,500 amu. For example, a compound detected at *m/z* 749.48341 had 9, 5, and 3 candidate formulae at mass tolerances of 2, 1, and 0.5 ppm, respectively. IFS comparison was used to determine the molecular formula among the candidate formulae, as shown in Figure 3.

Figure 3 shows one experimental IFS and five candidate formula theoretical IFSs. The experimental monoisotopic molecular ion peak (M) was detected at *m/z* 749.48341, and the enlarged experimental mass spectra near M + 1, M + 2, and M + 3 are shown in Figure 3A. The relative intensities of the isotope peaks were calculated from the elemental composition and abundance. The peak intensities in Figure 3 are relative to the intensity of the monoisotopic peak (100%). GMF generated the following five theoretical IFSs of the candidate formulae near M + 1, M + 2, and M + 3: [C_40_H_57_N_14_O]^+^ (749.48343 amu; Figure 3B), [C_42_H_69_O_11_]^+^ (749.48344 amu; Figure 3C), [C_42_H_62_N_8_O_3_Na]^+^ (749.48371 amu; Figure 3D), [C_39_H_66_N_8_O_4_K]^+^ (749.48386 amu; Figure 3E), and [C_55_H_61_N_2_]^+^ (749.48293 amu; Figure 3F). The monoisotopic peak of the molecular ion is a single peak by definition; other isotopic peaks such as M + 1, M + 2, and M + 3 can be attributed to heavy isotopic atom substitution and have fine structures. The M + 1 fine structures in Figure 3 indicate that the peak caused by a ^15^N substitution (Δm = 0.99703 amu) was separated from the peak caused by a substitution ^13^C (Δm = 1.00335 amu). The mass resolving power of 360,000 at *m/z* 750 was sufficient to resolve the ^15^N substitution peak from the ^13^C substitution peaks. Owing to the absence of the ^15^N-substituted isotopic peaks (~750.48 amu) in the experimental data, only the candidate formulae F and C remained. These two candidates can be distinguished by the relative intensities of the ^13^C-substituted isotopic peaks near M + 1 for formulae C (43.26%) and F (59.49%). The experimental peak intensity (~43%) near M + 1 can clearly select the true formula [C_42_H_69_O_11_]^+^ without ambiguity. A comparison of the M + 2 and M + 3 fine structures shows that the IFS of [C_42_H_69_O_11_]^+^ (Figure 3C) is the best fit to the compound at *m*/*z* 749.48341 due to the absence of ^15^N- and ^41^K-substituted isotopic peaks and the relatively high-intensity peaks caused by the presence of ^18^O substitutions. Thus, from the IFS comparison shown in Figure 3, the molecular formula of the compound detected at *m/z* 749.48341 is assigned to C_42_H_68_O_11_. IFS could differentiate Na adduct ions from K adduct ions, because single isotope elemental Na has no effect on IFS, whereas the ^40^K isotope causes a conspicuous M + 2 peak split, as shown in Figure 3E.

Differences in the elemental composition of two compounds can be deduced from the mass difference of two peaks measured with high mass accuracy [21]. As shown in the inset of Figure 2A, the mass differences between the peaks at *m/z* 441.37264 and 457.36756, and between those at *m/z* 457.36756 and 459.38323 are 15.99492 and 2.01567 amu, respectively, which are equivalent to mass differences with the addition of ^16^O_1_ and ^1^H_2_, respectively. Given that the molecular formula of the ion at *m/z* 441.37264 was found to be [C_30_H_49_O_2_]^+^, i.e., [C_30_H_48_O_2_ + H]^+^ by IFS, the molecular formulae of the peaks at *m/z* 457.36756 and 459.38323 were determined to be [C_30_H_48_O_3_ + H]^+^ and [C_30_H_50_O_3_ + H]^+^, respectively. This suggests that C_30_H_48_O_3_ is an oxidative derivative of C_30_H_48_O_2_, and C_30_H_50_O_3_ is a reductive derivative of C_30_H_48_O_3_. Formula determination by mass difference is useful for identifying a series of compounds with the same skeleton, as reported in petroleum analysis [22].

Using high mass accuracy, the exact mass difference, and a comparison of IFS, we determined the molecular formula of 123 compounds (of the 405 detected compounds), including 33 putative ginsenosides and their derivatives (Table 1). Note that determining the molecular formula does not definitively identify ginsenosides because some ginsenosides are structural isomers with identical molecular formulae. A molecule and its molecular formula can be identified from the structural isomers with further structural analysis methods such as tandem mass spectrometry (MS/MS) and nuclear magnetic resonance spectroscopy. The application of MS/MS analysis to the three selected extracted compounds is described below as an example. All molecular formulae of the 123 compounds are listed in Table 2. The molecular formulae of small molecules (<400 amu) were determined mainly by high mass accuracy with 0.5 ppm tolerance and typically yielded a single candidate, while for large molecules (>400 amu), IFS was required to select the correct formula from multiple candidates. Based on these results, improved mass accuracy and resolution could facilitate the characterization of large phytochemicals. The resolving power of 360,000 at 750 amu clearly showed the IFS in Figure 3, and IFSs near *m*/*z* 1000 were observed with the mass resolving power of 300,000 (data not shown). This result indicates that an accuracy of 0.5 ppm and a mass resolving power of 300,000 are required to use IFS for determining the molecular formulae of phytochemicals with <1,000 amu. Enhancing the sensitivity would improve the HT nature of this method because the weak intensities of the M + 2 and M + 3 isotope peaks remain as the main obstacle to determining molecular formulae.

**Table 1:** The ionic molecular formulae of the ginsenosides

**Molecular formula**	***m/z***		**Error (ppm)**	**R.T. (min)**
	**Observed**	**Theoretical**		
[C_37_H_57_O_6_]^+^	597.41497	597.41497	0.0	
[C_36_H_59_O_7_]^+^	603.42554	603.42553	0.0	
[C_36_H_61_O_7_]^+^	605.44122	605.44118	0.1	26.1, 34.1
[C_36_H_59_O_8_]^+^	619.42065	619.42045	0.3	
[C_36_H_61_O_8_]^+^	621.43612	621.43610	0.0	19.7
[C_36_H_59_O_9_]^+^	635.41538	635.41536	0.0	
[C_36_H_61_O_9_]^+^	637.43120	637.43101	0.3	
[C_36_H_63_O_9_]^+^	639.44671	639.44666	0.1	27.7
[C_38_H_43_O_9_]^+^	643.29022	643.29016	0.1	
[C_42_H_67_O_10_]^+^	731.47294	731.47287	0.1	
[C_41_H_69_O_11_]^+^	737.48366	737.48344	0.3	
[C_42_H_67_O_11_]^+^	747.46772	747.46779	0.1	
[C_42_H_69_O_11_]^+^	749.48341	749.48344	0.0	
[C_41_H_67_O_12_]^+^	751.46265	751.46270	0.1	
[C_41_H_69_O_12_]^+^	753.47851	753.47835	0.2	
[C_41_H_71_O_12_]^+^	755.49414	755.49400	0.2	
[C_42_H_67_O_12_]^+^	763.46280	763.46269	0.1	
[C_42_H_69_O_12_]^+^	765.47857	765.47835	0.3	
[C_42_H_71_O_12_]^+^	767.49423	767.49400	0.3	20.5, 27.4
[C_42_H_69_O_13_]^+^	781.47345	781.47327	0.2	
[C_42_H_71_O_13_]^+^	783.48893	783.48892	0.0	15.9, 23.1
[C_42_H_73_O_13_]^+^	785.50453	785.50457	0.0	29.6
[C_42_H_73_O_14_]^+^	801.49969	801.49948	0.3	20.6, 24.7
[C_42_H_70_O_14_Na] ^+^	821.46595	821.46578	0.2	
[C_47_H_79_O_17_]^+^	915.53129	915.53118	0.1	
[C_47_H_81_O_17_]^+^	917.54683	917.54683	0.0	
[C_48_H_81_O_17_]^+^	929.54693	929.54683	0.1	
[C_47_H_81_O_18_]^+^	933.54187	933.54174	0.1	19.5, 25.3
[C_48_H_83_O_18_]^+^	947.55748	947.55739	0.1	20.5, 27.4
[C_48_H_83_O_19_]^+^	963.55260	963.55231	0.3	23.0
[C_53_H_91_O_22_]^+^	1079.59959	1079.59965	0.1	26.3
[C_53_H_91_O_23_]^+^	1095.59467	1095.59457	0.1	22.4
[C_54_H_93_O_23_]^+^	1109.61042	1109.61022	0.2	25.2

Molecular formulae were determined using high mass accuracy and IFS comparison. Retention times were measured using LC/FT-ICR MS.

**Table 2:** The ionic molecular formulae of the ginseng extract compounds

**Molecular formula**	***m/z***		**Error (ppm)**	**Molecular formula**	***m/z***		**Error (ppm)**	**Molecular formula**	***m/z***		**Error (ppm)**	**Molecular formula**	***m/z***		**Error (ppm)**
	**Observed**	**Theoretical**			**Observed**	**Theoretical**			**Observed**	**Theoretical**			**Observed**	**Theoretical**	
[C_16_H_22_O_4_Na]^+^	301.14103	301.14103	0.0	[C_22_H_26_O_8_Na]^+^	441.15193	441.15199	0.1	[C_36_H_61_O_9_]^+^	637.43120	637.43101	0.3	[C_42_H_74_NO_12_]^+^	784.52052	784.52055	0.0
[C_15_H_28_NO_5_]^+^	302.19631	302.19620	0.4	[C_30_H_49_O_2_]^+^	441.37264	441.37271	0.2	[C_36_H_63_O_9_]^+^	639.44671	639.44666	0.1	[C_42_H_73_O_13_]^+^	785.50453	785.50457	0.0
[C_22_H_39_]^+^	303.30465	303.30463	0.1	[C_30_H_51_O_2_]^+^	443.38829	443.38836	0.1	[C_38_H_43_O_9_]^+^	643.29022	643.29016	0.1	[C_42_H_55_N_4_O_11_]^+^	791.38649	791.38619	0.4
[C_12_H_21_O_9_]^+^	309.11801	309.11801	0.0	[C_21_H_19_O_11_]^+^	447.09215	447.09219	0.1	[C_36_H_60_O_8_Na]^+^	643.41806	643.41804	0.1	[C_39_H_65_N_6_O_11_]^+^	793.47102	793.47058	0.5
[C_20_H_40_NO]^+^	310.31043	310.31044	0.0	[C_30_H_49_O_3_]^+^	457.36756	457.36762	0.1	[C_42_H_83_N_2_O_2_]^+^	647.64493	647.64493	0.0	[C_42_H_71_O_14_]^+^	799.48393	799.48383	0.1
[C_16_H_30_NO_5]_^+^	316.21188	316.21185	0.1	[C_30_H_51_O_3_]^+^	459.38323	459.38327	0.1	[C_36_H_63_O_10_]^+^	655.44161	655.44157	0.0	[C_42_H_73_O_14_]^+^	801.49969	801.49948	0.3
[C_22_H_41_O]^+^	321.31517	321.31519	0.1	[C_21_H_18_O_11_Na]^+^	469.07406	469.07413	0.2	[C_40_H_35_N_8_O_2_]^+^	659.28748	659.28775	0.4	[C_42_H_70_O_13_Na]^+^	805.47108	805.47086	0.3
[C_12_H_21_O_10_]^+^	325.11288	325.11292	0.1	[C_30_H_51_O_4_]^+^	475.37820	475.37819	0.0	[C_36_H_60_O_9_Na]^+^	659.41305	659.41295	0.0	[C_42_H_55_N_4_O_12_]^+^	807.38148	807.38110	0.5
[C_23_H_42_N]^+^	332.33115	332.33118	0.1	[C_36_H_53_O_3_]^+^	533.39895	533.39892	0.1	[C_38_H_45_O_10_]^+^	661.30079	661.30072	0.1	[C_42_H_72_O_13_Na]^+^	807.48670	807.48651	0.2
[C_22_H_42_NO]^+^	336.32600	336.32609	0.3	[C_31_H_58_NO_6_]^+^	540.42580	540.42587	0.1	[C_36_H_62_O_9_Na]^+^	661.42878	661.42860	0.3	[C_42_H_70_O_14_Na]^+^	821.46595	821.46578	0.2
[C_22_H_44_NO] ^+^	338.34169	338.34174	0.2	[C_24_H_47_O_13_]^+^	543.30113	543.30112	0.0	[C_38_H_42_O_9_Na]^+^	665.27219	665.27210	0.1	[C_42_H_72_O_14_Na]^+^	823.48149	823.48143	0.1
[C_19_H_22_O_5_Na] ^+^	353.13599	353.13594	0.1	[C_33_H_54_NO_5_]^+^	544.39968	544.33965	0.0	[C_44_H_87_N_2_O_2_]^+^	675.67620	675.67621	0.0	[C_47_H_58_N_6_O_6_Na]^+^	825.43096	825.43100	0.1
[C_42_H_48_N_12_]^2+^	360.20570	360.20570	0.0	[C_29_H_56_NO_8_]^+^	546.40007	546.40004	0.1	[C_35_H_64_O_11_Na]^+^	683.43424	683.43408	0.2	[C_47_H_79_O_17_]^+^	915.53129	915.53118	0.1
[C_22_H_43_NONa] ^+^	360.32366	360.32369	0.1	[C_36_H_55_O_4_]^+^	551.40959	551.40949	0.1	[C_43_H_50_N_6_O_2_Na]^+^	705.38859	705.38875	0.2	[C_47_H_81_O_17_]^+^	917.54683	917.54683	0.0
[C_25_H_46_N]^+^	360.36240	360.36248	0.2	[C_36_H_57_O_5_]^+^	569.42005	569.42005	0.0	[C_35_H_63_O_14_]^+^	707.42166	707.42123	0.5	[C_48_H_81_O_17_]^+^	929.54693	929.54683	0.1
[C_24_H_48_NO]^+^	366.37299	366.37304	0.1	[C_31_H_57_O_9_]^+^	573.39975	573.39971	0.1	[C_49_H_67_NONa]^+^	708.51151	708.51149	0.0	[C_47_H_81_O_18_]^+^	933.54187	933.54174	0.1
[C_26_H_39_O]^+^	367.29946	367.29954	0.2	[C_35_H_57_O_6_]^+^	573.41510	573.41497	0.2	[C_33_H_37_N_8_O_11_]^+^	721.25781	721.25763	0.3	[C_47_H_80_O_17_Na]^+^	939.52883	939.52877	0.1
[C_26_H_41_O_2_]^+^	385.31000	385.31011	0.3	[C_29_H_49_N_2_O_10_]^+^	585.33817	585.33817	0.0	[C_37_H_68_O_12_Na]^+^	727.46038	727.46030	0.1	[C_48_H_83_O_18_]^+^	947.55748	947.55739	0.1
[C_22_H_27_O_6_]^+^	387.18015	387.18022	0.2	[C_36_H_57_O_6_]^+^	585.41504	585.41497	0.0	[C_42_H_67_O_10_]^+^	731.47294	731.47287	0.1	[C_47_H_80_O_18_Na]^+^	955.52387	955.52369	0.2
[C_24_H_39_O_4_]^+^	391.28424	391.28429	0.1	[C_36_H_59_O_6_]^+^	587.43069	587.43062	0.1	[C_41_H_69_O_11_]^+^	737.48366	737.48344	0.3	[C_48_H_83_O_19_]^+^	963.55260	963.55231	0.3
[C_60_H_58_O_2_]^2+^	405.22149	405.22129	0.5	[C_40_H_62_NO_2_]^+^	588.47751	588.47751	0.0	[C_42_H_67_O_11_]^+^	747.46772	747.46779	0.1	[C_48_H_80_O_18_Na]^+^	967.52385	967.52369	0.2
[C_30_H_45_]^+^	405.35153	405.35158	0.1	[C_31_H_60_NO_9_]^+^	590.42630	590.42626	0.1	[C_42_H_69_O_11_]^+^	749.48341	749.48344	0.0	[C_48_H_82_O_18_Na]^+^	969.53944	969.53934	0.1
[C_30_H_47_]^+^	407.36722	407.36723	0.0	[C_35_H_59_O_7_]^+^	591.42553	591.42553	0.0	[C_41_H_67_O_12_]^+^	751.46265	751.46270	0.1	[C_43_H_76_N_6_O_17_Na]^+^	971.51569	971.51592	0.2
[C_22_H_26_O_6_Na]^+^	409.16212	409.16216	0.1	[C_37_H_57_O_6_]^+^	597.41497	597.41497	0.0	[C_41_H_69_O_12_]^+^	753.47851	753.47835	0.2	[C_50_H_89_N_5_O_12_Na]^+^	974.63999	974.63999	0.0
[C_24_H_38_O_4_Na]^+^	413.26618	413.26623	0.1	[C_36_H_59_O_7_]^+^	603.42554	603.42553	0.0	[C_41_H_71_O_12_]^+^	755.49414	755.49400	0.2	[C_48_H_82_O_19_Na]^+^	985.53436	985.53425	0.1
[C_20_H_34_NO_8_]^+^	416.22793	416.22789	0.1	[C_36_H_61_O_7_]^+^	605.44122	605.44118	0.1	[C_51_H_83_O_4_]^+^	759.62860	759.62859	0.0	[C_54_H_106_N_5_O_11_]^+^	1000.78821	1000.78834	0.1
[C_30_H_45_O]^+^	421.34643	421.34649	0.1	[C_33_H_61_O_10_]^+^	617.42597	617.42592	0.1	[C_42_H_67_O_12_]^+^	763.46280	763.46269	0.1	[C_53_H_91_O_22_]^+^	1079.59959	1079.59965	0.1
[C_30_H_47_O]^+^	423.36212	423.36214	0.0	[C_36_H_59_O_8_]^+^	619.42065	619.42045	0.3	[C_42_H_69_O_12_]^+^	765.47857	765.47835	0.3	[C_53_H_91_O_23_]^+^	1095.59467	1095.59457	0.1
[C_25_H_22_O_5_Na]^+^	425.13607	425.13594	0.3	[C_36_H_61_O_8_]^+^	621.43612	621.43610	0.0	[C_42_H_71_O_12_]^+^	767.49423	767.49400	0.3	[C_54_H_93_O_23_]^+^	1109.61042	1109.61022	0.2
[C_30_H_49_O]^+^	425.37777	425.37779	0.1	[C_33_H_64_NO_10_]^+^	634.45252	634.45247	0.1	[C_42_H_69_O_13_]^+^	781.47345	781.47237	0.2	[C_54_H_92_O_23_Na]^+^	1131.59222	1131.59216	0.1
[C_30_H_47_O_2_]^+^	439.35699	439.35706	0.2	[C_36_H_59_O_9_]^+^	635.41538	635.41536	0.0	[C_42_H_71_O_13_]^+^	783.48893	783.48892	0.0				

The molecular formulae were determined using a mass accuracy of 0.5 ppm and IFS observed by UHR ESI/ 15 T FT-ICR MS.

### LC/FT-ICR MS of ginseng extract

**Figure 4:**
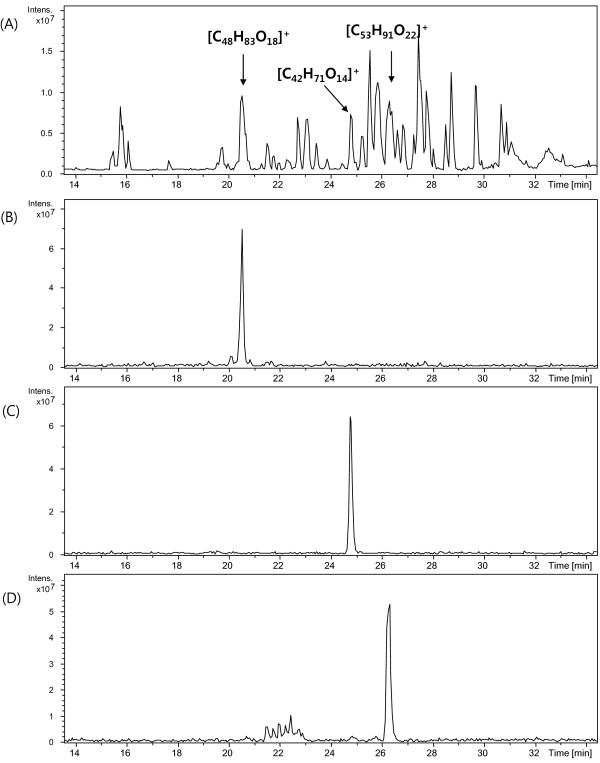
**Base peak chromatograms of LC/FT-ICR MS.** Base peak chromatograms of the ginseng extract (**A**) and the ginsenosides Re (**B**), Rf (**C**), and Rc (**D**). The molecular formulae of three extract compounds are labeled for comparison.

LC/FT-ICR MS of the extract was performed to compare the HT ability of UHR mass profiling and typical LC/MS analysis. The base peak chromatogram of the ginseng extract LC/FT-ICR MS is shown in Figure 4A. LC/FT-ICR MS did not provide the same observed mass accuracy as with UHR ESI/FT-ICR MS because its mass resolving power was about 55,000 at *m/z* 400 owing to the short transient time (0.29 s). The transient time of LC/FT-ICR MS was limited by the detection of eluted analytes with HPLC and the continuous introduction into the FT-ICR mass spectrometer. The LC/FT-ICR MS performed 733 scans in a 40-min run, and the two-scan accumulated LC/MS signals had very low S/N compared with the 20-scan accumulated signal with direct injection. The peaks in the 733 scans were counted as follows: all peaks with S/N >5 were sorted by *m/z* value, and peaks within a 0.5-ppm mass bin were combined into one peak with an averaged mass. The isobaric peaks with different retention times (T) (ΔT >1 min) were regarded as different peaks, although they were not discriminated and counted as a single peak in UHR ESI/FT-ICR MS.

After combining the 733 scans of the LC/FT-ICR MS experiment, 1,073 peaks were identified. Considering that isobaric peaks were disregarded, this suggests that many more compounds were detected by UHR ESI/FT-ICR MS. After deisotoping to remove electrical noise and minor chemicals whose isotope peaks were not detected, 129 compound peaks remained, which was far less than the 405 compounds detected by direct injection. These results suggest that UHR mass profiling is more efficient for multi-compound detection than LC/MS. This observation is not surprising because LC columns allow only a specific range of compounds to pass through based on the characteristics of the packed resin, whereas direct sample injection can deliver almost all of the compounds into the mass spectrometer. Molecular formula determination by IFS was not performed with LC/FT-ICR MS because of the low mass resolving power due to the shorter time-domain signal, which can also suppress the signal intensity. The molecular weight measured by the LC/FT-ICR mass spectrometer was used to assign a molecular formula, which had already been determined by UHR ESI/FT-ICR MS. Of the 33 putative ginsenoside compounds, 13 were detected by LC/FT-ICR MS. The molecular formulae and retention times of the observed putative ginsenoside compounds are listed in Table 1. The HT performance of LC/FT-ICR MS and UHR ESI FT-ICR MS is summarized in Table 3. LC/FT-ICR MS has the advantage of being able to separate isobaric compounds for distinguishing structural isomers. Furthermore, because LC/FT-ICR MS can separate constituent molecules and thereby reduce the ionization competition between numerous molecules with different charge affinities, as in direct injection mode, it allows the detection of extremely low-concentration molecules. On the other hand, UHR mass profiling by ESI/FT-ICR MS has the advantages of allowing the simultaneous analysis of the molecular formulae of multiple compounds in a single experiment and enabling the detection of very weak signals for as long as the sample exists, owing to its continuous accumulation of the ICR signal, which improves the S/N. The results of LC/FT-ICR MS provided more detailed analytical information, including retention time data; however, it seems certain that UHR mass profiling will be a competitive method in HT analysis due to its non-discriminative detection, higher sensitivity, and mass resolving power.

**Table 3:** Comparison of high-throughput performance between UHR ESI FT-ICR MS and LC/FT-ICR MS

	**UHR ESI FT-ICR MS**	**LC/FT-ICR MS**
Run Time	3.5 min	40 min
No. of peaks (S/N > 5)	1,552	1,073
No. of compounds	405	129
No. of ginsenosides	33	19^*^
Molecular formulas by IFS	123	0

* Six ginsenosides were detected at two different retention times.

To validate the molecular formulae determined in this study, three commercial ginsenosides (Rc (C_53_H_90_O_22_), Re (C_48_H_82_O_18_), Rf (C_42_H_70_O_14_)) and the ginseng extract were analyzed by LC/FT-ICR MS using the same experimental parameters. As shown in Figrue 4, three extract compounds at 20.5 s, 24.7 s, and 26.3 s showed the same retention time and molecular weight as Re, Rf, and Rc, respectively, indicating that these compounds are the commercial ginsenosides.

### MS/MS of ginseng extract

**Figure 5:**
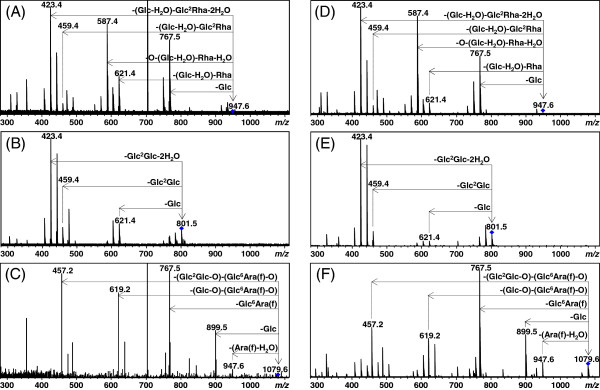
**CID MS/MS spectra of the three extract compounds and three commercial ginsenosides.** The compound (*m/z* 947.6) at 20.5 min (**A**), the compound (*m/z* 801.6) at 24.7 min (**B**), the compound (*m/z* 1,079.6) at 26.3 min (**C**), and the ginsenosides Re (947.6 amu) (**D**), Rf (801.5 amu) (**E**), and Rc (1079.6 amu) (**F**). The parent ion is labeled with a diamond. Note that the peaks at *m/z* 354.4 and 702.5 were electrical noise. Fragment peaks are annotated using reported rules [23]. The superscripts 2 and 6 denote the attachment position of the terminal sugar molecule. Glc = β-d-glucose, C_6_H_12_O_6_, 180 amu; Ara(f) = α-l-arabinofuranose, C_5_H_10_O_5_, 150 amu; Rha = α-l-rhamnose, C_6_H_12_O_5_, 164 amu.

For further validation, collision-induced dissociation (CID) tandem mass spectrometry (MS/MS) was performed on the three extract compounds to allow a comparison with MS/MS spectra of the three standard ginsenosides using LC/FT-ICR MS/MS. Using the *m*/*z* values and retention times obtained from LC/FT-ICR MS, the three extract compounds were selected as precursor ions for MS/MS at their retention times. For example, the *m*/*z* of the precursor ion at 20.5 min was 947.6. The MS/MS spectra of the three extract compounds and three standard ginsenosides are shown in Figure 5. The MS/MS spectra of the three extract compounds (Figure 5A– 5C) display relatively weak intensities and consequently are missing several minor fragments when compared with the three standard ginsenoside spectra (Figure 5D– 5F). Nevertheless, the overall fragmentation patterns of the compounds at 20.5, 24.7, and 26.3 min are quite similar to those of Re, Rf, and Rc, respectively [23]. The comparison of the MS/MS fragmentation pattern indicates that the three ginsenosides are correctly identified and, as a result, the other molecular formulae could also be accurately determined by UHR mass profiling.

## Conclusions

Ginseng ethanol extracts were analyzed using an UHR 15 T FT-ICR mass spectrometer. The resolution of the mass spectra in broadband mode was 1,000,000–270,000 at the range of *m/z* 290–1,100, which is sufficient to obtain the IFS of most compounds within that mass range. The HT performance of UHR ESI/FT-ICR MS was investigated by comparison with LC/FT-ICR MS for the same extract. The number of ginseng compounds detected by UHR ESI/FT-ICR MS was 405, which was more than three times the number detected by LC/FT-ICR MS. HT molecular formula determination of compounds in the ginseng extract was achieved using the formula-specific IFS and high mass accuracy. The molecular formulae of 123 compounds, including 33 ginsenosides, were accurately determined by a single mass spectrum. The molecular formula determined by UHR mass profiling was validated by a comparison of the CID fragmentation patterns and LC/FT-ICR MS retention times of three selected ginseng compounds containing standard responsive ginsenosides. In this study, UHR ESI/FT-ICR MS was able to detect a wide range of components in comparison with conventional LC/MS and an absolute determination of the molecular formula by IFS. UHR mass profiling may be very useful in studies of multi-component mixtures such as plant extracts and metabolomes owing to its unique ability to simultaneously determine molecular formulae.

## Methods

All MS was performed using a 15 T FT-ICR mass spectrometer (ApexQe, Bruker Daltonics, Billerica, MA, USA). UHR ESI/FT-ICR MS was used to profile extract compounds and determine the molecular formulae of the compounds, and LC/FT-ICR MS was employed to confirm the formulae determined by UHR mass profiling. Three known ginsenosides were analyzed to validate the molecular formulae determined by UHR ESI/FT-ICR MS.

### Samples

Korean ginseng (*Panax ginseng*) was purchased from the Korea Ginseng Corp (Daejeon, Korea). Dried and powdered roots (10 g) were dissolved in 500 mL of 70% ethanol for 2 days at room temperature with slow stirring, passed through filter paper (No. 3; Whatman, Maidstone, Kent, UK), and centrifuged at 6,000 *g* for 20 min to remove insoluble material. The supernatant was lyophilized to yield a powdered 70% ethanol extract. All chemicals used in this study were analytical grade and purchased from Sigma-Aldrich (St. Louis, MO, USA) unless otherwise noted.

### UHR ESI/FT-ICR MS

Ginseng extract powder (1 mg) was dissolved in 100 mL of 50% methanol, 0.1% formic acid (FA) aqueous solution and directly introduced into the 15 T FT-ICR mass spectrometer without separation steps, using a TriVersa NanoMate (Advion BioSciences, Ithaca, NY, USA) with a flow rate of approximately 400 nL/min for ESI. The MS parameters of the positive ESI mass spectrometer were an ESI voltage of 1500 V, mass range of *m/z* 250–2500, drying gas flow rate of 2.5 L/min, drying gas temperature of 190°C, skimmer voltage of 17 V, collision gas energy of −2.0 V, accumulation time of 1.0 s, transient length of 2.31 s, acquisition size of 4 MB, and a scan number of 20, with a sine-bell apodization window function applied in the time-domain signal. The base pressure in the ICR region of the instrument was 1.0 × 10^-9^ mbar, as measured using an ion gauge (370 Granville-Phillips, Helix Technology, Longmont, CO, USA), when the vacuum chamber was isolated from the magnet. External calibration was performed with quadratic regression using a 10 μg/mL arginine solution. All data were processed by DataAnalysis (ver. 3.4), an FT-ICR MS data processing program (Bruker Daltonics).

### LC/FT-ICR MS

The 70% ethanol extract components were analyzed by LC/FT-ICR MS using a high-performance liquid chromatography (HPLC) system (HP1200: Agilent, Santa Clara, CA, USA) and the 15 T FT-ICR mass spectrometer. Ginseng extract (1 mg) was dissolved in 1 mL of 50% methanol solution with 30 min of sonification. Extract solution (100 μL) was injected onto a C18 reverse-phase HPLC column (150 × 4.6 mm, 4 μm, 8 nm ODS-H80, YMC, Kyoto, Japan). A binary mobile phase was composed of solvents A (95:5 water/acetonitrile 0.1% FA) and B (95:5 acetonitrile/water 0.1% FA) and was applied to the column at a flow rate of 1 mL/min. The column temperature was set to 35°C. The solvent gradient was 5% B for 0–2 min, 10% B for 3 min, 50% B for 20 min, 100% B for 10 min, 100% B for 2 min, 5% B for 2 min, 5% B for 1 min; the run time was 40 min. The eluent was split 10:1 to produce a flow rate of 91 μL/min and to obtain positive ESI LC/MS data. MS parameters were an ESI capillary voltage of 4900 V, nebulizer gas rate of 2.5 L/min, drying gas flow rate of 3.5 L/min, drying gas temperature of 200°C, mass range of *m/z* of 250–4000, skimmer voltage of 15 V, collision gas energy of −2.0 V, accumulation time of 0.3 s, acquisition size of 512 KB, transient domain of 0.29 s, and an averaged scan number of 2, with a sine-bell function applied as an apodization window prior to the Fourier transform. CID was performed at the hexapole collision cell with Ar gas. The Ar collision energy and flow were set to −8.0 V and 0.33 L/h, respectively. The inflow of Ar was monitored by the pressure change from 4.1 × 10^-6^ to 5.3 × 10^-6^ mbar, which was measured by ion gauge 1 in Figure 1. Using the retention time and *m*/*z* obtained from the LC/FT-ICR MS experiment, the duty cycle of LC/MS/MS was set to 50%.

### Molecular formula determination

The molecular formula of a compound detected by UHR FT-ICR MS was determined by comparison of the theoretical and observed IFSs. The theoretical IFSs of candidate molecular ions were generated using the GMF utility of DataAnalysis (Bruker Daltonics). C, H, N, O, Na, and K were considered during the theoretical IFS calculation. Since Na and K were considered as adduct ions, the maximum numbers of Na and K were set equal to the charge number. Any candidate with more Na and K atoms than the charge number was removed from the candidate list. For singly charged ions, any candidate with both Na and K was removed from the candidate list. The parameters for the GMF calculation were a mass tolerance of 0.5 ppm, maximum H/C of 3, and an even electron configuration. The resolution of the theoretical IFSs of candidates generated by GMF was equal to the observed resolution, allowing for direct comparison of experimental and theoretical IFSs. The mass and abundance of the isotopes used in the theoretical mass calculations were obtained from the National Institute of Standards and Technology [24].

## Abbreviations

CID: Collision-induced dissociation; ESI: Electrospray ionization; FA: Formic acid; FT-ICR MS: Fourier transform ion cyclotron resonance mass spectrometry; HPLC: High-performance liquid chromatography; HR MS: High-resolution mass spectrometry; HT: High throughput; IFS: Isotopic fine structure; LC: Liquid chromatography; MS/MS: Tandem mass spectrometry; S/N: Signal-to-noise ratio; UHR: Ultra-high resolution.

## Competing interests

The authors declare that they have no competing interests.

## Authors’ contributions

KHP participated in the design of the study, drafted the manuscript, undertook data interpretation, and participated in performing the UHR ESI FT-ICR MS, LC/FT-ICR MS, and MS/MS experiments. MSK and SJB participated in the UHR ESI FT-ICR MS experiments and data processing. IHB participated in the LC/FT-ICR MS and MS/MS experiments. SWS, JK, and YKS participated in preparing the ginseng extract. YML participated in experimental design and coordination. HSK proposed the application of UHR mass spectrometry for the high-throughput determination of molecular formulae of natural products and completed the manuscript. All authors have read and approved the final manuscript.

## Supplementary Material

Additional file 1 **Theoretical isotopic fine structures of the candidate molecular ions in Figure **3**.** Click here for file
