# Supplementary material for: Simultaneous molecular formula determinations of natural compounds in a plant extract using 15 T Fourier transform ion cyclotron resonance mass spectrometry
Source: Plant Methods. 2013 May 30;9:15. doi: 10.1186/1746-4811-9-15 (PMC3706383; doi:10.1186/1746-4811-9-15)
Supplement: Additional file 1 — Theoretical isotopic fine structures of the candidate molecular ions in Figure 3. [file 1746-4811-9-15-S1.docx]

# Additional file 1

Theoretical isotopic fine structures of the candidate molecular ions in Figure 3.

| Formula | Class | Isotope | Mass | Abundance | Rel. Abun. |
| --- | --- | --- | --- | --- | --- |
| [C_40_H_57_N_14_O]^+^ | [M+1]^+^ | ^15^N_1_ | 750.480463 | 5.17 | 0.1195 |
|  |  | ^13^C_1_ | 750.486783 | 43.26 | 1.0000 |
|  |  | ^17^O_1_ | 750.487645 | 0.04 | 0.0009 |
|  |  | ^2^H_1_ | 750.489705 | 0.66 | 0.0153 |
|  | [M+2]^+^ | ^15^N_2_ | 751.477498 | 0.12 | 0.0132 |
|  |  | ^13^C_1_^15^N_1_ | 751.483818 | 2.24 | 0.2456 |
|  |  | ^2^H_1_^15^N_1_ | 751.486740 | 0.03 | 0.0033 |
|  |  | ^18^O_1_ | 751.487674 | 0.21 | 0.0230 |
|  |  | ^13^C_2_ | 751.490138 | 9.12 | 1.0000 |
|  |  | ^13^C_1_^17^O_1_ | 751.491000 | 0.02 | 0.0022 |
|  |  | ^13^C_1_^2^H_1_ | 751.493060 | 0.28 | 0.0307 |
|  | [M+3]^+^ | ^13^C_1_^15^N_2_ | 752.480853 | 0.05 | 0.0400 |
|  |  | ^13^C_2_^15^N_1_ | 752.487173 | 0.47 | 0.3760 |
|  |  | ^13^C_1_^18^O_1_ | 752.491029 | 0.09 | 0.0720 |
|  |  | ^13^C_3_ | 752.493493 | 1.25 | 1.0000 |
|  |  | ^13^C_2_^2^H_1_ | 752.496415 | 0.06 | 0.0480 |
| [C_42_H_69_O_11_]^+^ | [M+1]^+^ | ^13^C_1_ | 750.486794 | 45.43 | 1.0000 |
|  |  | ^17^O_1_ | 750.487656 | 0.42 | 0.0092 |
|  |  | ^2^H_1_ | 750.489716 | 0.79 | 0.0174 |
|  | [M+2]^+^ | ^18^O_1_ | 751.487685 | 2.26 | 0.2244 |
|  |  | ^13^C_2_ | 751.490149 | 10.07 | 1.0000 |
|  |  | ^13^C_1_^17^O_1_ | 751.491011 | 0.19 | 0.0189 |
|  |  | ^13^C_1_^2^H_1_ | 751.493071 | 0.36 | 0.0358 |
|  | [M+3]^+^ | ^13^C_1_^18^O_1_ | 752.491040 | 1.03 | 0.7103 |
|  |  | ^13^C_3_ | 752.493504 | 1.45 | 1.0000 |
|  |  | ^2^H_1_^18^O_1_ | 752.493962 | 0.02 | 0.0138 |
|  |  | ^2^H_2_^17^O_1_ | 752.494366 | 0.04 | 0.0276 |
|  |  | ^13^C_2_^2^H_1_ | 752.496425 | 0.08 | 0.0552 |
| [C_42_H_62_N_8_Na_1_O_3_]^+^ | [M+1]^+^ | ^15^N_1_ | 750.480743 | 2.95 | 0.0649 |
|  |  | ^13^C_1_ | 750.487063 | 45.43 | 1.0000 |
|  |  | ^17^O_1_ | 750.487925 | 0.11 | 0.0024 |
|  |  | ^2^H_1_ | 750.489985 | 0.71 | 0.0156 |
|  | [M+2]^+^ | ^15^N_2_ | 751.477778 | 0.04 | 0.0040 |
|  |  | ^13^C_1_^15^N_1_ | 751.484098 | 1.34 | 0.1331 |
|  |  | ^2^H_1_^15^N_1_ | 751.487020 | 0.02 | 0.0020 |
|  |  | ^18^O_1_ | 751.487954 | 0.62 | 0.0616 |
|  |  | ^13^C_2_ | 751.490418 | 10.07 | 1.0000 |
|  |  | ^13^C_1_^17^O_1_ | 751.491280 | 0.05 | 0.0050 |
|  |  | ^13^C_1_^2^H_1_ | 751.493340 | 0.32 | 0.0318 |
|  | [M+3]^+^ | ^13^C_1_^15^N_2_ | 752.481133 | 0.02 | 0.0138 |
|  |  | ^15^N_1_^18^O_1_ | 752.484989 | 0.02 | 0.0138 |
|  |  | ^13^C_2_^15^N_1_ | 752.487453 | 0.30 | 0.2069 |
|  |  | ^13^C_1_^18^O_1_ | 752.491309 | 0.28 | 0.1931 |
|  |  | ^13^C_3_ | 752.493773 | 1.45 | 1.0000 |
|  |  | ^13^C_2_^2^H_1_ | 752.496695 | 0.07 | 0.0483 |
| [C_39_H_66_KN_8_O_4_]^+^ | [M+1]^+^ | ^15^N_1_ | 750.480895 | 2.95 | 0.0699 |
|  |  | ^13^C_1_ | 750.487215 | 42.18 | 1.0000 |
|  |  | ^17^O_1_ | 750.488077 | 0.15 | 0.0036 |
|  |  | ^2^H_1_ | 750.490137 | 0.76 | 0.0180 |
|  | [M+2]^+^ | ^15^N_2_ | 751.477930 | 0.04 | 0.0046 |
|  |  | ^41^K_1_ | 751.481980 | 7.22 | 0.8328 |
|  |  | ^13^C_1_^15^N_1_ | 751.484250 | 1.25 | 0.1442 |
|  |  | ^2^H_1_^15^N_1_ | 751.487172 | 0.02 | 0.0023 |
|  |  | ^18^O_1_ | 751.488106 | 0.82 | 0.0946 |
|  |  | ^13^C_2_ | 751.490570 | 8.67 | 1.0000 |
|  |  | ^13^C_1_^17^O_1_ | 751.491432 | 0.06 | 0.0069 |
|  |  | ^13^C_1_^2^H_1_ | 751.493492 | 0.32 | 0.0369 |
|  | [M+3]^+^ | ^41^K_1_^15^N_1_ | 752.479015 | 0.21 | 0.0691 |
|  |  | ^15^N_1_^18^O_1_ | 752.485141 | 0.02 | 0.0066 |
|  |  | ^13^C_1_^41^K_1_ | 752.485334 | 3.04 | 1.0000 |
|  |  | ^13^C_2_^15^N_1_ | 752.487605 | 0.26 | 0.0855 |
|  |  | ^2^H_1_^41^K_1_ | 752.488256 | 0.05 | 0.0164 |
|  |  | ^13^C_1_^18^O_1_ | 752.491461 | 0.35 | 0.1151 |
|  |  | ^13^C_3_ | 752.493925 | 1.16 | 0.3816 |
|  |  | ^13^C_2_^2^H_1_ | 752.496847 | 0.07 | 0.0230 |
| [C_55_H_61_N_2_]^+^ | [M+1]^+^ | ^15^N_1_ | 750.479961 | 0.74 | 0.0124 |
|  |  | ^13^C_1_ | 750.486281 | 59.49 | 1.0000 |
|  |  | ^2^H_1_ | 750.489203 | 0.70 | 0.0118 |
|  | [M+2]^+^ | ^13^C_1_^15^N_1_ | 751.483316 | 0.44 | 0.0253 |
|  |  | ^13^C_2_ | 751.489636 | 17.37 | 1.0000 |
|  |  | ^13^C_1_^2^H_1_ | 751.492558 | 0.42 | 0.0242 |
|  | [M+3]^+^ | ^13^C_2_^15^N_1_ | 752.486671 | 0.13 | 0.0392 |
|  |  | ^13^C_3_ | 752.492990 | 3.32 | 1.0000 |
|  |  | ^13^C_2_^2^H_1_ | 752.495912 | 0.12 | 0.0361 |

- Abundance: The intensities of related isotope peaks are listed when the peak intensity of M^+^ is assigned to be 100.
- Rel. Abun.: The relative intensities of the isotopic fine peaks around M+1, M+2, M+3 were normalized for each group around M+1, M+2, or M+3.
